# Evaluating Compliance and Care Pathways in a Section 136 Suite: A Retrospective Audit

**DOI:** 10.1192/bjo.2026.11745

**Published:** 2026-06-30

**Authors:** Arshpreet Singh Badesha, Raghu Vutla

**Affiliations:** South West Yorkshire Partnership Teaching NHS Foundation Trust, Wakefield, United Kingdom

## Abstract

**Aims:**

The Section 136 (s136) suite provides a safe and controlled environment for individuals detained under the Mental Health Act (MHA), enabling timely mental health assessment whilst reducing stigma and distress. This audit assessed the s136 suite at Fieldhead Hospital in Wakefield. The audit evaluated compliance with local standard operating procedures (SoPs), including timeliness of MHA assessments, involvement of the Intensive Home Based Treatment Team (IHBTT), documentation, and communication with general practitioners (GPs).

**Methods:**

A retrospective audit was conducted of adult patients admitted to the Wakefield s136 suite between September 2024 and September 2025. Data were extracted from SystmOne records. Inclusion criteria were adult patients admitted within the audit period; patients whose records were inaccessible were excluded. Standards assessed included patient demographics, community mental health team involvement, time from referral to assessment, MHA assessment outcomes, involvement of IHBTT, documentation of MHA assessments, and communication of outcomes to GPs

**Results:**

A total of 111 patients were identified, of whom 100 met inclusion criteria. Sixty percent of presentations occurred out of hours, and 55% of patients were known to Community Mental Health Teams (CMHTs), with nearly 60% of these under Enhanced Teams. While 60% of patients were assessed within four hours and 90% within twelve hours, IHBTT provided face-to-face input in only 45% of cases. Documentation compliance was high, with 99% of assessments recorded by Section 12 doctors. However, communication to GPs was low, with over 70% of discharged patients lacking documented GP correspondence.

**Conclusion:**

The audit demonstrates good compliance with assessment timeliness and documentation standards but highlights significant gaps in IHBTT involvement and GP communication. Improved gatekeeping through consistent IHBTT face-to-face attendance, enhanced relapse prevention planning for patients under community teams, and mandatory communication of assessment outcomes to GPs are recommended to improve adherence to SoPs and continuity of care.

